# Treatment gaps and potential cardiovascular risk reduction from expanded statin use in the US and England

**DOI:** 10.1371/journal.pone.0190688

**Published:** 2018-03-21

**Authors:** Peter Ueda, Thomas Wai-Chun Lung, Yuan Lu, Joshua A. Salomon, Kazem Rahimi, Philip Clarke, Goodarz Danaei

**Affiliations:** 1 Department of Global Health and Population, Harvard School of Public Health, Boston, Massachusetts, United States of America; 2 Clinical Epidemiology Unit, Department of Medicine, Solna, Karolinska Institutet, Stockholm, Sweden; 3 Department of Global Health Policy, Graduate School of Medicine, The University of Tokyo, Tokyo, Japan; 4 The George Institute for Global Health, Camperdown, Australia; 5 Yale/ Yale-New Haven Hospital, Center for Outcomes Research and Evaluation (CORE), New Haven, Connecticut, United States of America; 6 The George Institute for Global Health, Oxford Martin School, University of Oxford, Oxford, United Kingdom; 7 Centre for Health Policy, School of Population and Global Health, The University of Melbourne, Carlton, Victoria, Australia; 8 Department of Epidemiology, Harvard School of Public Health, Boston, Massachusetts, United States of America; University of Bologna, ITALY

## Abstract

**Background:**

The updated national guidelines for cardiovascular risk assessment and lipid modification in the UK and US expand the indications for statin therapy in primary prevention to adults with moderate risk of cardiovascular disease (CVD) but many adults at high CVD risk remain untreated in both countries. We set out to identify treatment gaps in English and American adults at moderate and high risk of cardiovascular disease (CVD), and to estimate the number of CVD events that would be prevented from expanding statin therapy to those who are currently untreated.

**Methods:**

We used nationally representative samples of 10,375 English adults and 7,687 US adults aged 40–75 years and free of existing CVD from the Health Survey for England 2009–2013, and the National Health and Nutrition Examination Survey 2007–2012 in the US. We used the risk algorithms and the risk thresholds for statin therapy recommended by each country’s national guideline to categorize the survey participants into moderate-risk (≥10% to <20% 10-year risk of CVD in England and ≥7.5% to <20% risk in the US) or high-risk (≥20%risk) and simulated the number of events that would be prevented from expansion of statin therapy to those currently untreated.

**Results:**

Close to half of adults at high CVD risk in England (46.0%) and the US (49.7%) were not receiving statins. Expanding statin use to 1.45 million high-risk adults in England would save 101,000 (95% CI = 81,000–120,000) CVD events in the next 10 years compared with 128,000 (103,000–154,000) CVD events that would be prevented from expanding treatment to 3.64 million untreated moderate-risk adults. In the US, expanding statin use to 5.27 million untreated high-risk adults would save 384,000 (305,000–461,000) CVD events over 10 years compared with 616,000 (493,000–738,000) CVD events that would be prevented from treating 20.29 million untreated moderate-risk adults.

**Conclusions:**

In both England and the US, expanding statin therapy to untreated moderate-risk adults would prevent a comparable number of events as expanding statin use to a much smaller number of currently untreated high-risk adults. A large potential for CVD prevention remains from improving coverage of statin therapy among high-risk adults.

## Introduction

Recent releases of updated national guidelines for cardiovascular risk assessment and lipid modification in the US and the UK have been accompanied by considerable controversy.[1–5] The focus of this controversy has been on the lowered risk thresholds for statin treatment in patients with no previous cardiovascular disease (CVD). In the US, the 2013 guidelines of the American College of Cardiology and the American Heart Association (ACC-AHA)[6] recommend statin therapy for primary prevention in those with a ≥7.5% 10-year CVD risk as compared with the threshold of 20% 10-year risk of coronary heart disease used in the Third Adult Treatment Panel (ATP-III) guidelines. [7,8] In the UK, the threshold was lowered from 20% 10-year CVD risk in the 2008 NICE guidelines to ≥10% risk in the updated guidelines released in 2014. [9,10]

The lowered risk thresholds for statin therapy followed recent meta-analyses of clinical trials showing that statins similarly reduced CVD risk across a wide range of cardiovascular risk profiles, and that serious adverse effects, with the exception of a small increase in risk of diabetes, were rare.[11,12] Since statins are inexpensive and CVD causes substantial health consequences and healthcare costs, it was considered cost-effective to expand the indications for statin therapy. Critics of the new guidelines argue that the chances of benefiting from statins for patients at CVD risk levels around the new treatment thresholds are too small to justify the risk of adverse effects, which may be more frequent in the general population than in clinical trials, and the huge burden that the millions of newly eligible patients would place on the healthcare system.[5,13,14]

There is no dispute, however, that individuals at high risk of CVD would benefit the most from statins. Yet, a substantial number of these patients are currently not receiving statins in both US and UK [15–17] and the resultant burden of preventable CVD events has not yet been rigorously quantified. More importantly, it is not clear how the potential impact of increasing treatment coverage among high-risk individuals would compare with that of initiating treatment in those at moderate risk, as suggested by the new guidelines.

We used data from the National Health and Nutrition Examination Surveys (NHANES) in the US and the Health Survey of England (HSE) to estimate the number of CVD events in each country that could be prevented from increasing coverage of statin treatment among adults at high CVD risk, as compared with expanding treatment to moderate-risk adults.

## Methods

### Study population

We used data from NHANES and HSE collected before the introduction of the updated guidelines; the NHANES rounds from 2007 to 2012, and the HSE samples from 2009 to 2013. In each round, NHANES and HSE recruit a representative sample of the non-institutionalized U.S. population, and the general population in England, respectively. Although the NICE guidelines recommend cardiovascular risk assessment and statin therapy in ages up to 84 years, we limited the analyses to ages 40 to 75 years because the ACC-AHA guidelines only apply to this age range. To estimate 10-year CVD risk, we used the pooled cohort equations in NHANES, and the QRISK2 equation[18,19] in HSE as suggested by the respective countries’ guidelines.

Of the 9,610 adults aged 40–75 years who were part of the medical examination sample in NHANES, we excluded 1,092 participants with self-reported CVD (myocardial infarction, angina, or stroke) and 830 participants who had missing data on any of the risk factors included in the ACA-AHA Pooled cohorts risk prediction equations (age, sex, race, total cholesterol, HDL cholesterol, systolic blood pressure, blood pressure lowering medication, diabetes and smoking status), and one participant who had no information on statins. Our final study population in NHANES included 7,687participants.

Of the 12,463 participants aged 40–75 years who were part of the blood test sample in the HSE, we excluded 399 participants with self-reported CVD (coronary heart disease, angina pectoris or stroke) and 1,689 participants who had missing data on any of the risk factors included in our application of the QRISK2 equation[18,19] (age, sex, ethnicity, smoking status, body mass index, systolic blood pressure, blood pressure lowering medication, and total-to-HDL cholesterol ratio). Our final study population included 10,375 HSE participants. HSE does not provide information on several variables used in the QRISK2 equation. These are atrial fibrillation, rheumatoid arthritis, chronic kidney disease (stage 4 or 5), type 1 diabetes, Townsend deprivation score, and cardiovascular disease in a 1^st^ degree relative before the age 60 years. We set the values of these variables to “no” or “0”.

The Cholesterol Treatment Trialists’ (CTT) meta-analysis has showed that the absolute risk reduction achieved by statin treatment depends on both the degree of low density lipoprotein (LDL)-cholesterol lowering and the underlying CVD risk of the patient.[20] In primary prevention, each 1 mmol/L reduction in LDL-cholesterol resulted in a 25% (95% confidence interval [CI] 20%–30%) proportional decrease in the risk of CVD.[11] Because correct estimation of LDL-cholesterol requires fasting blood samples and triglyceride measurements that were not available in the HSE sample, we assumed a 1 mmol/L (38.6 mg/dL) reduction in LDL-cholesterol for all patients.[21] In sensitivity analyses described below, we used the fasting blood sample in NHANES to account for pretreatment LDL-cholesterol.

### Statistical analysis

We assessed three scenarios in each of the surveys separately. First, we calculated the individual-level 10-year CVD risk under the current treatment based on the participants’ risk factor levels (current treatment scenario). We then estimated the 10-year CVD risk under no treatment for participants who reported receiving statins by multiplying their current risk with the inverse of the risk reduction conferred by statins (no treatment scenario). We then estimated the 10-year CVD risk in the populations if moderate- and high-risk participants had received treatment by multiplying the estimated risk for participants who did not receive statins with the risk reduction that would have been achieved from statin treatment (full treatment scenario).

We categorized the populations into three groups according to their 10-year CVD risk under no treatment: low (<10%), moderate (7.5% to <20%), and high risk (≥20%). We used the 7.5% risk as the cut-off for moderate risk according to the ACC-AHA guidelines, and considered a 10-year risk of ≥20% to be ‘high’ as it is a threshold used in the 2008 NICE guidelines[10] and in several national and international guidelines.[22,23] We also performed the analyses using the 10% cut-off for moderate risk according to the 2014 NICE guidelines.

We used the two survey samples and corresponding sampling weights to estimate the number of people that would fall into each of the CVD risk categories in the US and England national population. We used a population of 111.3 million US adults[24] and 21.5 million English adults aged 40 to 75 years without a history of CVD. We counted the number of untreated (statin-naïve) individuals by risk group under the no treatment scenario, and predicted the number of CVD events as estimated by the corresponding risk scores that would occur within 10 years in each scenario described above and repeated the analyses by sex and age group (40–59 and 60–75 years). The number of CVD events that could be prevented in the full treatment scenario compared with current treatment coverage in each risk group depends on three factors: the proportion of population in the risk group; the current coverage of statins; and the estimated individual-level 10-year CVD risk among participants.

We illustrated the potential gains in risk reduction from expansion of treatment in the currently statin-naïve moderate and high-risk individuals by ranking the participants by their level of 10-year CVD risk under the different treatment scenarios.

For each survey and treatment scenario, we estimated uncertainty of the predicted number of CVD events due to sampling variability of the survey and uncertainty in the effect of statins using a simulation approach. We drew repeatedly from the survey population while accounting for sample weights. The risk reduction from statin treatment was drawn from an independent log-normal distribution. We used 1000 draws, and report 95% confidence intervals based on the resulting distributions of the number of predicted CVD events.

The predicted CVD risks of the two risk scores are not comparable because the QRISK2 equation includes non-fatal coronary heart disease, angina pectoris and transient ischemic attack which are not included in the Pooled cohorts equations. [18,25] Therefore, we also performed the analyses using the Globorisk,[26] which is a risk score for fatal-and-nonfatal cardiovascular disease that is recalibrated for use in different countries.

To account for the fact that the reduction in LDL-cholesterol from statin therapy is proportional to the pretreatment LDL-cholesterol levels,[20] we also performed sensitivity analyses using data from the fasting blood sample in NHANES. We used the LDL-cholesterol concentrations as calculated by the Friedewald formula in 3,010 statin naïve individuals with a triglyceride level of <400mg/dL. We estimated the LDL-reduction from statin therapy by assuming a 43% reduction in LDL-cholesterol concentration which is typically produced by atorvastatin 20mg daily[27]—the currently recommended first-line treatment in primary prevention.[9] We then used the approach presented by Soran et al.[20], and estimated the risk reduction from statin therapy by multiplying the CVD risk with the statin risk ratio (per 1 mmol LDL- reduction) to the power of the LDL- reduction. We did not perform these analyses in HSE because pretreatment LDL-levels were not available.

Analyses were performed in Stata (version 12.0, Stata Corporation, College Station. (TX). Institutional review board approval was not needed for this study because we used secondary data from NHANES and HSE.

## Results

Under the no treatment scenario (had nobody been treated with statins), 27.85 million US adults (25.0%) were categorized as moderate-risk (median current 10-year CVD risk using pooled cohorts equations of 10.9%) according to the ACC-AHA guidelines, and another 10.60 million adults (9.5%) were at high risk (median risk 24.4%). 20.29 million (72.9%) of the moderate-risk adults, and 5.27 million (49.7%) of the high-risk adults did not report receiving statins. (Table 1)

**Table 1:** Cardiovascular risk profile of adults aged 40–75 years without existing cardiovascular disease (CVD) and at moderate and high risk of CVD in England using HSE (2009–2013) and in the US using NHANES (2007–2012).

Population characteristics*	US		England	
	Moderate-risk** (≥7.5% to <20%)	High-risk (≥20%)	Moderate-risk** (≥10% to <20%)	High-risk (≥20%)
Number (% of total population)	27,845 (25.0)	10,600 (9.5)	4,657 (21.7)	3,157 (14.7)
Female sex	10,086 (36.2)	3,236 (30.5)	1,913 (41.1)	845 (26.8)
Age	61 (55–66)	69 (62–73)	63 (58–67)	68 (63–72)
Ethnicity				
White or not stated (England)/ Non-Hispanic white or other (USA)	21,721 (78.0)	7,823 (73.8)	4,465 (95.9)	3,011 (95.4)
South Asian (England)/Hispanic (USA)	2,619 (9.4)	1,158 (10.9)	122 (2.6)	109 (3.5)
Other ethnicities*** (England)/Black (USA)	3,505 (12.6)	1,619 (15.3)	70 (1.5)	36 (1.1)
Higher education†	6,728 (24.2)	1,919 (18.1)	860 (18.5)	416 (13.2)
Health insurance††	24,071 (86.4)	9,723 (91.7)		
Household income†††				
Lowest tertile (England) / <20,000 USD (USA)	3,943 (14.2)	1,964 (18.5)	1,148 (24.6)	1,045 (33.1)
Middle tertile (England)/20,000 to <75,000 USD (USA)	13,823 (49.6)	6,017 (56.8)	1,431 (30.7)	1,029 (32.6)
Highest tertile (England)/>75,000 USD (USA)	9,178 (33.0)	2,344 (22.1)	1,322 (28.4)	556 (17.6)
Total cholesterol (mmol/L)	5.3 (4.7–6.1)	4.9 (4.2–5.7)	5.7 (4.9–6.5)	5 (4.2–5.9)
HDL-cholesterol (mmol/L)	1.2 (1.0–1.5)	1.2 (1.0–1.4)	1.4 (1.2–1.7)	1.2 (1.0–1.5)
Systolic blood pressure (mmHg)	127 (118–138)	138 (126–150)	133 (122–144)	136.5 (126–148.5)
Diabetes	5,304 (19.0)	5,424 (51.2)	423 (9.1)	1,175 (37.2)
Current smoking	7,534 (27.1)	2,931 (27.7)	930 (20.0)	736 (23.3)
Receiving blood pressure medication	11,479 (41.2)	6,709 (63.3)	1,248 (26.8)	1,691 (53.6)
Receiving lipid therapy	7,553 (27.1)	5,329 (50.3)	1,017 (21.8)	1,704 (54.0)
Current 10-year cardiovascular disease risk ϕ	10.9 (8.6–13.9)	24.4 (20.6–31.2)	13.1 (11.2–15.6)	23.9 (20.6–29.5)

* Number (in thousands rounded to nearest 1000) and % of population reported for categorical characteristics and median (interquartile range) for continuous ones.

**Risk groups are categorized according to 10-year CVD risk. The 7.5% risk threshold is recommended in the AHA-ACC guidelines in the US for statin therapy for primary prevention, and the 10% risk threshold is recommended in the 2014 NICE guidelines in the UK.

*** Including Chinese, African, Caribbean or any other Black/African/Caribbean background, Arab, or any other ethnic group

^†^College degree or higher in the US, and NVQ4/NVQ5/Degree or equivalent in England.

^††^ Applicable for the US only

^†††^Total household income used in the US, and equivalised household income tertiles for the whole population in England. Proportions do not add up to 100% because 3.4% of the US population and 16.5% of the English population had missing information on household income.

^ϕ^ Pooled cohorts equations were used in NHANES and QRISK2 was used in HSE to estimate 10-year CVD risk.

In England, 4.66 million adults (21.7%) were categorized as moderate CVD risk (median current 10-year CVD risk using QRISK2 = 13.1%), and another 3.16 million adults (14.7%) were at high risk (median risk 23.9%). 3.64 million (78.2%) of the moderate-risk adults, and 1.45 million (46.0%) of the high-risk adults were not receiving statins. (Table 1)

**Fig 1:**
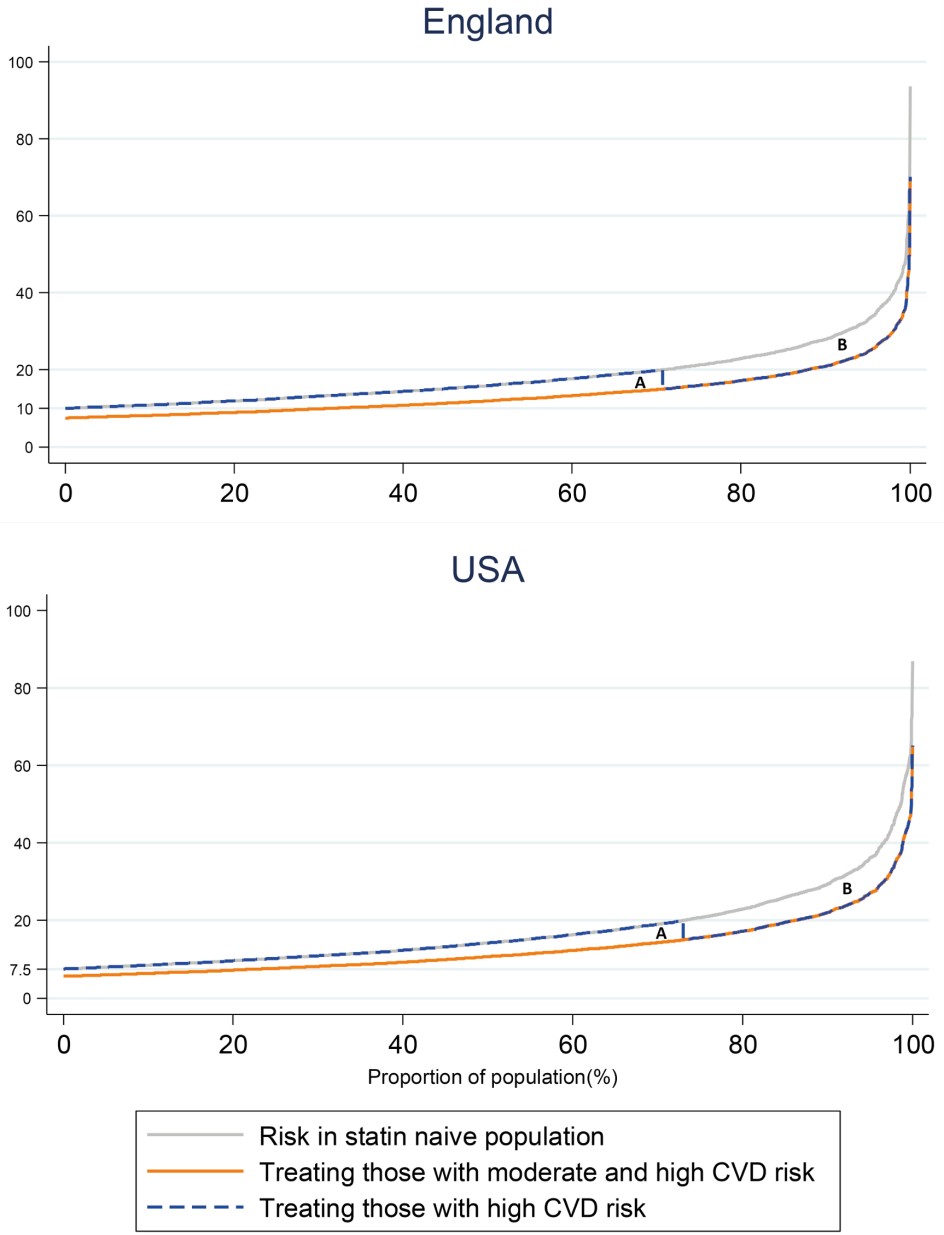
Distribution of 10 year CVD risk in currently statin-naïve US and English population aged 40–75 years at moderate or high risk of CVD. The dotted grey line represents the current risk distribution in the population; the dotted orange line represents the risk distribution that would be achieved from treating both moderate- and high-risk individuals with statins; and the blue line shows the risk distribution if individuals with high CVD risk were treated with statins. Area A represents the risk reduction achieved from treatment of statin-naïve individuals at moderate risk; area B represents the risk reduction that could be achieved from treatment of statin-naïve individuals at high-risk. Moderate risk is ≥7.5% to <20% in the US, and ≥10% to <20% in England. High risk is ≥20% in both countries.

In the US, expansion of treatment to 20.3 million currently untreated moderate-risk individuals (using the 7.5% threshold) would prevent 616,000 (493,000–738,000) CVD events (NNT of 33 (27–41)), whereas treating the 5.2 million currently untreated high-risk individuals would prevent another 384,000 (305,000–461,000) CVD events (NNT of 14 (11–17)) (Table 2 and Fig 1). When treating both the moderate and the high- risk group, NNT was 25 (21–32).

**Table 2:** Cardiovascular disease (CVD) events over 10 years (in thousands and rounded to nearest 1000 with 95% confidence intervals) in adults aged 40–75 years without existing CVD in in the US using NHANES (2007–2012) and in England using HSE (2009–2013).

	Risk group*	Population		Number of CVD events over 10 years		
		Total	Under no treatment	Had nobody received statins	Prevented by current statin coverage	Preventable by full statins coverage per guidelines
All adults	**US**					
	Moderate	27,845 (26,629–28,903)	20,293 (19,817–20,953)	3,427 (3,376–3,469)	240 (187–288)	616 (493–738)
	High	10,600 (9,849–11,580)	5,271 (4,791–5,688)	3,318 (3,210–3,423)	446 (329–583)	384 (305–461)
	**England**					
	Moderate	4,657 (4,459–4,846)	3,640 (3,522–3,743)	668 (662–673)	38 (32–44)	128 (103–154)
	High	3,157 (2,964–3,370)	1,453 (1,355–1,557)	954 (928–982)	138 (103–177)	101 (81–120)
40–59 years	**US**					
	Moderate	12,321 (11,555–13,161)	9,478 (9,048–9,911)	1,402 (1,372–1,428)	83 (61–106)	268 (214–320)
	High	1,651 (1,374–2,028)	998 (812–1,155)	479 (447–511)	46 (29–67)	74 (58–90)
	**England**					
	Moderate	1,504 (1,405–1,617)	1,098 (1,042–1,140)	202 (199–205)	14 (11–18)	36 (29–43)
	High	421 (351–483)	171 (142–207)	126 (120–133)	19 (14–25)	12 (10–15)
60–75 years	**US**					
	Moderate	15,524 (14,764–16,074)	10,815 (10,446–11,316)	2,025 (1,991–2,056)	158 (123–186)	348 (279–417)
	High	8,949 (8,325–9,704)	4,273 (3,869–4,632)	2,839 (2,737–2,939)	400 (297–522)	310 (248–372)
	**England**					
	Moderate	3,152 (2,993–3,302)	2,542 (2,449–2,645)	466 (461–470)	24 (20–27)	92 (74–110)
	High	2,736 (2,577–2,906)	1,283 (1,194–1,368)	828 (803–854)	119 (88–153)	88 (71–105)
Men	**US**					
	Moderate	17,759 (16,984–18,658)	13,628 (13,185–14,038)	2,208 (2,166–2,239)	136 (107–166)	416 (332–498)
	High	7,364 (6,781–8,070)	3,866 (3,498–4,192)	2,320 (2,237–2,407)	297 (215–391)	283 (226–339)
	**England**					
	Moderate	2,744 (2,608–2,896)	2,219 (2,125–2,289)	398 (393–402)	20 (17–23)	79 (64–95)
	High	2,312 (2,159–2,462)	1,099 (1,022–1,185)	718 (700–743)	101 (76–131)	78 (63–93)
Women	**US**					
	Moderate	10,086 (9,254–10,700)	6,664 (6,371–7,129)	1,220 (1,193–1,249)	104 (77–125)	200 (161–241)
	High	3,236 (2,823–3,781)	1,404 (1,177–1,618)	998 (949–1,044)	148 (109–197)	101 (80–122)
	**England**					
	Moderate	1,913 (1,788–2,016)	1,421 (1,369–1,485)	270 (266–273)	18 (14–22)	49 (39–59)
	High	845 (758–954)	354 (304–396)	235 (226–243)	37 (27–48)	22 (18–27)

* Risk groups are categorized according to 10-year CVD risk under no treatment (had nobody been treated with statins). Moderate risk is ≥7.5% to <20% in the US, and ≥10% to <20% in England. High risk is ≥20% in both countries.

Age-specific analyses in the US showed that 10.82 million (53.3%) of statin-naïve moderate-risk adults and 348,000 (279,000–417,000; 56.6%) of the CVD events that would be prevented by expansion to full treatment in this risk group were between ages 60 and 75 years. The same age group contributed to 81.1% of the statin-naïve adults and 80.7% of the CVD events that would be prevented under treatment expansion among high-risk individuals. (Table 2) Sex-specific analyses showed that 13.63 million (67.2%) of the statin-naïve moderate-risk adults and 3.87 million (73.3%) of the statin-naïve high-risk adults were men. Of the number of CVD events that would be prevented from expansion to full treatment among moderate-risk adults, 67.5% were in men, and among high-risk adults 73.3% were in men. (Table 2)

In England expansion of statin therapy to 3.64 million untreated moderate-risk individuals would prevent 128,000 (103,000–154,000) CVD events (Number Needed to Treat or NNT of 28 (24–34) whereas treating 1.45 million untreated high-risk individuals would prevent 101,000 (81,000–120,000) CVD events (NNT of 14 (12–18)) (Table 2 and Figure). When treating both moderate and high-risk groups NNT was 24 (19–29).

When stratified by age, we found that among 60 to 75 years olds in England, expansion of treatment to 2.54 million statin-naïve moderate-risk adults (69.8% of all moderate-risk individuals in this age) would prevent 92,000 (74,000–110,000) CVD events over 10 years, which is 71.9% of all events that would be prevented among moderate-risk adults 40 years old or older under expansion of statins. Similarly, 88% of the treatment naïve high-risk individuals were 60 to 75 years old and 87% of the potentially preventable CVD events occurred among this age group. (Table 2) Sex-specific analyses showed that 2.22 million (61.0%) of the statin-naïve moderate-risk adults and 1.10 million (75.6%) of the statin-naïve high-risk adults were men. Of the number of CVD events that would be prevented from expansion to full treatment among moderate-risk adults, 61.7% were in men, and among high-risk adults 77.2% were in men. (Table 2)

The untreated high-risk adults constituted 4.7% of the US population aged 40–75 years and free of CVD, and 6.8% of the English population (Table 3). The corresponding numbers for the untreated moderate-risk adults were 18.2% and 17.0%. In both the US and England, the untreated high-risk adults were more likely to belong to the lowest income groups. A substantial proportion had diabetes (45.2% in US, and 27.9% in England), or were under blood pressure treatment (50.9% in US, and 37.0% in England).

**Table 3:** Cardiovascular risk profile of statin-naïve adults aged 40–75 years without existing cardiovascular disease (CVD) and at moderate and high risk of CVD in the US using NHANES (2007–2012), and in England using HSE (2009–2013).

Population characteristics*	US		England	
	Moderate-risk** (≥7.5% to <20%)	High-risk (≥20%)	Moderate-risk** (≥10% to <20%)	High-risk (≥20%)
Number, (% of total population)	20,293 (18.2)	5,271 (4.7)	3,640 (17.0)	1,453 (6.8)
Female sex	6,664 (32.8)	1,404 (26.6)	1,421 (39)	354 (24.4)
Age	60 (54–66)	68 (61–72)	63 (58–68)	69 (64–73)
Ethnicity				
White or not stated (England)/ Non-Hispanic white or other (USA)	15,449 (76.1)	3,944 (74.8)	3,518 (96.6)	1,412 (97.1)
South Asian (England)/Hispanic (USA)	2,125 (10.5)	600 (11.4)	82 (2.3)	38 (2.6)
Other ethnicities*** (England)/Black (USA)	2719 (13.4)	727 (13.8)	40 (1.1)	4 (0.3)
Higher education†	4632 (22.8)	953 (18.1)	703 (19.3)	175 (12.0)
Health insurance††	16,995 (83.8)	4,633 (87.9)		
Household income†††				
Lowest tertile (England) / <20,000 USD (USA)	3,066 (15.1)	1,065 (20.2)	846 (23.2)	470 (32.4)
Middle tertile (England)/20,000 to <75,000 USD (USA)	10,372 (51.1)	3,014 (57.2)	1,134 (31.2)	471 (32.4)
Highest tertile (England)/≥75,000 USD (USA)	6,195 (30.5)	1,038 (19.7)	1,049 (28.8)	249 (17.1)
Total cholesterol (mmol/L)	5.6 (4.9–6.2)	5.5 (4.8–6.4)	5.9 (5.3–6.6)	5.8 (5.2–6.6)
HDL-cholesterol (mmol/L)	1.2 (1.0–1.5)	1.1 (0.9–1.4)	1.4 (1.2–1.8)	1.2 (1.0–1.5)
Systolic blood pressure (mmHg)	129 (119–140)	141 (129–155)	133.5 (122.5–145)	138.5 (127.5–151.5)
Diabetes	3,291 (16.2)	2,384 (45.2)	214 (5.9)	406 (27.9)
Current smoking	6,332 (31.2)	1,886 (35.8)	777 (21.3)	398 (27.4)
Receiving blood pressure medication	7,106 (35.0)	2,684 (50.9)	809 (22.2)	537 (37.0)
Current 10-year cardiovascular disease risk^ϕ^	11.4 (9.1–14.8)	26.5 (22.3–32.4)	13.7 (11.7–16.5)	25.3 (22.3–30.6)

* Number (in thousands rounded to nearest 1000) and % of population reported for categorical characteristics and median (interquartile range) for continuous ones.

**Risk groups are categorized according to 10-year CVD risk. The 7.5% risk threshold is recommended in the AHA-ACC guidelines in the US for statin therapy for primary prevention, and the 10% risk threshold is recommended in the 2014 NICE guidelines in the UK.

*** Including Chinese, African, Caribbean or any other Black/African/Caribbean background, Arab, or any other ethnic group

^†^College degree or higher in the US and NVQ4/NVQ5/Degree or equivalent in England.

^††^ Applicable for USA only

^†††^ Total household income was used in the US, and equivalised household income tertiles for the whole population in England. Proportions do not add up to 100% because 3.4% of the US population, and 16.5% of the English population had missing information on household income.

^ϕ^ Pooled cohorts equations were used in NHANES and QRISK2 was used in HSE to estimate 10-year CVD risk.

Using the 10%10-year CVD risk cut-off for moderate risk in the US and the 7.5% cut-off for moderate risk in England, did not considerably change the results (S1 Table). In sensitivity analyses using Globorisk, the predicted 10-year CVD risks were lower than those estimated using QRISK2 (in England) and, Pooled cohorts equations (in the US) which have previously been shown to overestimate risks in contemporary populations,[1,28] leading to a much smaller number of CVD events prevented by full statin coverage in the high-risk category compared with the moderate-risk category (S2 Table). However, the NNT remained similar: 36 [30–45] for moderate-risk and 15 [12–19] for high-risk in US adults, and 30 [25–37] for moderate-risk and 16 [13–20] for high-risk in English adults.

When we used the fasting sample in NHANES to account for pretreatment LDL-cholesterol levels in the estimation of risk reduction from statin treatment, the number of CVD events that would be prevented from treating statin naïve adults was 790,000 (645,000–929,000; NNT = 24 [21–30]) in moderate-risk and 479,000 (386,000–576,000; NNT = 10 [8–12]) in high-risk adults (S3 Table). Although the estimated risk reduction from statin therapy was larger, the relative number of CVD events in high-risk versus moderate-risk adults was similar to those observed in our main analysis.

## Discussion

We found that almost half of the adults at high CVD risk in the US and England are not receiving statins. Increasing statin uptake to these 5.27 million adults in the US will save 384,000 CVD events in the next 10 years compared with 616,000 CVD events that would be prevented from initiating treatment in 20.29 million moderate-risk adults, as suggested by the ACC-AHA guidelines. In England, expanding statin coverage to 1.45 million untreated high-risk individuals would save 101,000 CVD events over 10 years compared with 128,000 CVD events that would be prevented from expanding statin use to 3.64 million untreated moderate-risk adults as recommended by the 2014 NICE guidelines. There was a larger number of untreated high-risk individuals among older adults (60 to 75 years old) and among men in both countries, especially in England.

The low treatment rates in high-risk adults observed in our study are similar to those observed in previous analyses. In the UK, 69% of the patients at high risk (as estimated without accounting for ongoing statin therapy) recorded in the Clinical Practice Research Datalink between 2007 and 2011 were not receiving statins,[17] and an analysis of the NHANES showed that 40% of US adults who would be eligible for statin treatment under the ATPIII-guidelines remained untreated.[15]

Considering the substantial differences between the healthcare systems in the US and England, the similarities of the findings regarding treatment gaps point to similar underlying problems. Although some patients may remain untreated due to specific reasons such as statin intolerance or personal preference,[29] it is clear that major improvements in coverage of statin therapy among high-risk adults are feasible. Many physicians still prescribe statins based solely on cholesterol levels or patient characteristics rather than the patient’s predicted CVD risk.[16,17,30–33] Uptake of risk-based prescription of statins can be improved by training physicians,[34] and by providing tools (such as risk charts and computer-based applications) that make implementation of risk-based screening in clinical settings easier. Moreover, although many patients initially experience minor side effects or discontinue treatment,[35,36] they can eventually take up statin treatment.[36,37] Other strategies to improve adherence to treatment include using non-physician clinicians,[38] mHealth technologies[39] and financial incentives to patients and doctors.[40] Finally, targeted strategies for finding and treating high-risk individuals, for example by pre-selecting patients who are likely to have a high CVD risk using routinely available information and inviting them for a full risk assessment, are feasible and effective.[41–43] We found that many of the untreated high-risk adults were already identified by the healthcare system as they had either diabetes or hypertension. In addition, approximately 6 million US adults who have a history of CVD and 380,000 English adults with the same history are currently not under statin treatment.[15,16] These patients are easy to identify, and would clearly benefit from statins.

A full evaluation of the potential for CVD prevention from treating high- versus moderate-risk adults would require comparing rates of both successful treatment initiation and adherence to medications. If these factors were considered, the relative number of preventable CVD events among untreated high-risk versus moderate-risk adults may be larger than in our analyses because adherence to statin treatment tends to be lower among patients with fewer CVD risk factors,[29,44,45] and uptake of statins may be lower among moderate-risk individuals.[45,46] While the lower NNT in the high risk group provide some evidence that it is more cost-effective to treat high risk individuals, this would need to be considered alongside factors such identifying patients that require treatment and the relative compliance in both groups. Hence future studies are needed to compare the cost-effectiveness of strategies for finding and treating patients at different levels of CVD risk.

Strengths of the study include the use of nationally representative data, and uncertainty analyses accounting for sampling variability of the surveys and uncertainty in the effect of statins. Because the predicted CVD risks in our main analyses for the US and England are not directly comparable, we also performed the same analyses using Globorisk, recalibrated for US and UK separately. Although Globorisk predicted substantially lower CVD risks in both countries, and a higher relative number of preventable events in moderate- versus high-risk adults, the treatment rates and NNT in the high-risk adults were comparable to those estimated using the local models.

Our study has a number of limitations. First, we applied an assumed 25% risk reduction from statin therapy, corresponding to a 1 mmol/L lowering of LDL-cholesterol even though the degree of LDL-lowering depends on pretreatment LDL-levels. Our sensitivity analyses using the NHANES fasting blood sample showed that the relative number of preventable events in high- versus moderate-risk adults were similar to those in the main analysis. Second, the effect of statins in the clinical trials may not be generalizable to the general population of US or England even though the protective effect of statins on CVD is consistent across levels of CVD risk, and demographic factors.[11,21,47] Third, the Pooled cohort equations provided by the ACC-AHA guidelines overestimated the 10-year CVD risk when applied in other US cohorts[1,25,28] and we may therefore have overestimated the preventable number of CVD events in the US. In fact, in our sensitivity analysis using Globorisk only 3.0% of the US population were categorized as high-risk under the no treatment scenario compared with 9.5% using the Pooled cohorts equations. Fourth, prevalence of existing CVD may have been underreported in HSE because only the 2011 survey included questions about lifetime CVD whereas the other rounds let the participant list longstanding illnesses. Fifth, due to the age range included in the ACC-AHA guidelines, the upper age limit of our study is 75 years. There is a need to explore treatment rates and preventable CVD events in elderly populations, particularly as a large population-based study has shown a diminishing propensity to prescribe statins with both increasing age and cardiovascular risk.[48] Finally, although we accounted for sampling variability of the surveys and uncertainty in the effect of statins, our uncertainty analysis did not include uncertainty in risk prediction.

In conclusion, we found that there are almost four times as many statin-naïve moderate-risk (≥7.5% to <20% 10-year CVD risk) adults as there are statin-naïve high-risk adults in the US, and more than 2.5 times as many statin-naïve moderate-risk (≥10% to <20% 10-year CVD risk) adults as high-risk adults in England. Expanding statin treatment to untreated individuals would prevent comparable numbers of events in the moderate-risk and high-risk adults in both countries. There is no dispute that high-risk adults would benefit the most from statin therapy, and these findings show the large potential for CVD prevention that remains from improving coverage of treatment in this group of patients in both England and the US.

## Supporting information

S1 Table Cardiovascular disease (CVD) events (in thousand with 95% confidence intervals) over 10 years in adults aged 40–75 years without existing CVD under different treatment scenarios by 10-year CVD risk group the 2014 NICE guidelines definition of moderate risk in the US, and using the ACC-AHA definition of moderate risk in England. NHANES data were used for US, and HSE data were used for England.
(DOCX)

S2 Table CVD events over 10 years (in thousands and rounded to nearest 1000 with 95% confidence intervals) in adults aged 40–75 years without existing CVD under different treatment scenarios using Globorisk in the US using NHANES (2007–2012), and in England using HSE (2009–2013). (DOCX)

S3 Table Cardiovascular disease (CVD) events prevented over 10 years (in thousands and rounded to nearest 1000 with 95% confidence intervals) in statin-naïve adults aged 40–75 years without existing CVD and triglyceride levels <400 mg/dL under full treatment in the US using the fasting sample (n = 3,010) in NHANES (2007–2012) and accounting for pretreatment LDL-cholesterol levels. (DOCX)
