# Supplementary material for: Treatment gaps and potential cardiovascular risk reduction from expanded statin use in the US and England
Source: PLoS One. 2018 Mar 21;13(3):e0190688. doi: 10.1371/journal.pone.0190688 (PMC5862405; doi:10.1371/journal.pone.0190688)
Supplement: S1 Table — NHANES data were used for US, and HSE data were used for England. (DOCX) [file pone.0190688.s001.docx]

**S1 Table. Cardiovascular disease (CVD) events (in thousand with 95% confidence intervals) over 10 years in adults aged 40-75 years without existing CVD under different treatment scenarios by 10-year CVD risk group the 2014 NICE guidelines definition of moderate risk in the US, and using the ACC-AHA definition of moderate risk in England. NHANES data were used for US, and HSE data were used for England.**

|  | Risk group* | Population | | CVD events over 10 years under 3 scenarios | | |
| --- | --- | --- | --- | --- | --- | --- |
|  |  | Total | Statin-naïve | Had nobody received statins | Prevented by current statin coverage | Preventable by full statins coverage  per guidelines |
| Total | **US** |  |  |  |  |  |
|  | Moderate (≥10% to <20%) | 18,347 (17,395-19,198) | 13,091 (12,717-13,608) | 2,607 (2,574-2,633) | 191 (147-225) | 461 (370-550) |
|  | High (≥20%) | 10,600 (9,827-11,536) | 5,271 (4,770-5,690) | 3,318 (3,203-3,414) | 446 (330-579) | 384 (306-459) |
|  | **England** |  |  |  |  |  |
|  | Moderate (≥7.5% to <20%) | 6,612 (6,397-6,814) | 5,397 (5,285-5,514) | 837 (830-846) | 43 (35-49) | 167 (134-200) |
|  | High (≥20%) | 3,157 (2,965-3,355) | 1,453 (1,357-1,559) | 954 (928-981) | 138 (102-176) | 101 (80-120) |
| 40-59 years | **US** |  |  |  |  |  |
|  | Moderate (≥10% to <20%) | 6,815 (6,196-7,366) | 5,116 (4,894-5,426) | 927 (910-946) | 59 (43-72) | 173 (139-207) |
|  | High (≥20%) | 1,651 (1,351-2,001) | 998 (825-1,156) | 479 (448-511) | 46 (30-67) | 74 (58-90) |
|  | **England** |  |  |  |  |  |
|  | Moderate (≥7.5% to <20%) | 2,699 (2,569-2,828) | 2,142 (2,081-2,196) | 305 (301-311) | 18 (14-22) | 59 (47-70) |
|  | High (≥20%) | 421 (350-484) | 171 (142-207) | 126 (121-133) | 19 (14-25) | 12 (10-15) |
| 60-75 years | **US** |  |  |  |  |  |
|  | Moderate (≥10% to <20%) | 11,532 (10,877-12,134) | 7,975 (7,613-8,387) | 1,680 (1,653-1,701) | 133 (102-158) | 288 (231-345) |
|  | High (≥20%) | 8,949 (8,351-9,702) | 4,273 (3,859-4,650) | 2,839 (2,728-2,930) | 400 (296-518) | 310 (247-371) |
|  | **England** |  |  |  |  |  |
|  | Moderate (≥7.5% to <20%) | 3,914 (3,755-4,072) | 3,255 (3,157-3,366) | 532 (526-538) | 25 (21-28) | 108 (86-129) |
|  | High (≥20%) | 2,736 (2,593-2,897) | 1,283 (1,195-1,368) | 828 (804-854) | 119 (88-152) | 88 (70-105) |
| Men | **US** |  |  |  |  |  |
|  | Moderate (≥10% to <20%) | 12,009 (11,243-12,676) | 8,853 (8,537-9,278) | 1,709 (1,681-1,731) | 115 (88-137) | 312 (250-372) |
|  | High (≥20%) | 7,364 (6,769-8,040) | 3,866 (3,478-4,204) | 2,320 (2,235-2,400) | 297 (218-390) | 283 (227-338) |
|  | **England** |  |  |  |  |  |
|  | Moderate (≥7.5% to <20%) | 3,785 (3,635-3,936) | 3,161 (3,064-3,246) | 488 (482-495) | 22 (19-26) | 100 (80-120) |
|  | High (≥20%) | 2,312 (2,160-2,453) | 1,099 (1,027-1,184) | 718 (700-741) | 101 (75-130) | 78 (62-93) |
| Women | **US** |  |  |  |  |  |
|  | Moderate (≥10% to <20%) | 6,338 (5,781-6,894) | 4,238 (3,977-4,534) | 899 (877-915) | 76 (56-94) | 149 (119-179) |
|  | High (≥20%) | 3,236 (2,811-3,759) | 1,404 (1,152-1,611) | 998 (945-1,042) | 148 (108-197) | 101 (80-121) |
|  | **England** |  |  |  |  |  |
|  | Moderate (≥7.5% to <20%) | 2,828 (2,696-2,947) | 2,235 (2,178-2,312) | 349 (344-354) | 21 (16-24) | 67 (53-80) |
|  | High (≥20%) | 845 (760-947) | 354 (309-399) | 235 (225-245) | 37 (27-47) | 22 (18-27) |

*According to 10-year CVD risk had nobody been treated with statins
