# Supplementary material for: Treatment gaps and potential cardiovascular risk reduction from expanded statin use in the US and England
Source: PLoS One. 2018 Mar 21;13(3):e0190688. doi: 10.1371/journal.pone.0190688 (PMC5862405; doi:10.1371/journal.pone.0190688)
Supplement: S2 Table — (DOCX) [file pone.0190688.s002.docx]

**S2 Table. CVD events over 10 years (in thousands and rounded to nearest 1000 with 95% confidence intervals) in adults aged 40-75 years without existing CVD under different treatment scenarios using Globorisk in the US using NHANES (2007-2012), and in England using HSE (2009-2013).**

|  | Risk group* | Population | | CVD events over 10 years under 3 scenarios | | |
| --- | --- | --- | --- | --- | --- | --- |
|  |  | Total | Statin-naïve | Had nobody received statins | Prevented by current statin coverage | Preventable by full statins coverage  per guidelines |
| Total | **US** |  |  |  |  |  |
|  | Moderate | 26,938 (25,825-28,142) | 17,711 (17,066-18,326) | 3,052 (3,013-3,091) | 277 (214-345) | 486 (392-581) |
|  | High | 3,352 (2,781-3,969) | 1,582 (1,311-1,888) | 888 (857-925) | 115 (78-156) | 107 (84-129) |
|  | **England** |  |  |  |  |  |
|  | Moderate | 2749 (2,595-2,884) | 1,660 (1,592-1,746) | 374 (370-378) | 38 (29-46) | 55 (44-66) |
|  | High | 659 (556-761) | 222 (183-267) | 174 (170-180) | 29 (21-38) | 14 (11-17) |
| 40-59 years | **US** |  |  |  |  |  |
|  | Moderate | 10,694 (9,982-11,465) | 7,892 (7,451-8,279) | 1,139 (1,115-1,162) | 77 (56-101) | 207 (167-248) |
|  | High | 881 (663-1130) | 631 (504-740) | 258 (232-285) | 17 (8-29) | 48 (36-60) |
|  | **England** |  |  |  |  |  |
|  | Moderate | 393 (333-457) | 239 (208-270) | 51 (49-52) | 5 (3-7) | 7.7 (6.2-9) |
|  | High | 46 (27-69) | 16 (7-26) | 12 (11-13) | 2 (1-3) | 1.1 (0.8-1.4) |
| 60-75 years | **US** |  |  |  |  |  |
|  | Moderate | 16,244 (15,561-16,876) | 9,819 (9,367-10,285) | 1,913 (1,881-1,946) | 200 (153-246) | 279 (224-333) |
|  | High | 2,471 (2,008-2,965) | 951 (736-1,200) | 630 (609-657) | 98 (69-131) | 59 (47-71) |
|  | **England** |  |  |  |  |  |
|  | Moderate | 2,356 (2,233-2,465) | 1,420 (1,365-1,495) | 323 (320-327) | 33 (26-40) | 48 (38-57) |
|  | High | 613 (517-709) | 206 (167-250) | 162 (158-168) | 27 (20-35) | 13 (10-16) |
| Men | **US** |  |  |  |  |  |
|  | Moderate | 17,117 (16,306-17,979) | 11,718 (11,198-12,198) | 1,933 (1,901-1,964) | 161 (124-202) | 322 (259-385) |
|  | High | 1,805 (1,394-2,218) | 880 (697-1,104) | 469 (444-498) | 57 (35-79) | 61 (47-74) |
|  | **England** |  |  |  |  |  |
|  | Moderate | 1,808 (1,704-1,919) | 1,068 (1,012-1,130) | 246 (243-249) | 26 (20-32) | 36 (28-42) |
|  | High | 418 (333-500) | 145 (113-184) | 107 (104-111) | 18 (12-23) | 9 (7-11) |
| Women | **US** |  |  |  |  |  |
|  | Moderate | 9,821 (9,154-10,479) | 5,993 (5,635-6,346) | 1,119 (1,092-1,144) | 116 (88-145) | 164 (131-196) |
|  | High | 1,547 (1,223-1,918) | 702 (521-863) | 419 (395-447) | 59 (40-81) | 46 (36-56) |
|  | **England** |  |  |  |  |  |
|  | Moderate | 941 (845-1,025) | 591 (551-644) | 128 (126-131) | 12 (9-15) | 20 (16-24) |
|  | High | 241 (195-290) | 77 (57-98) | 67 (64-71) | 12 (9-15) | 5 (3.9-6) |

* According to 10-year CVD risk had nobody been treated with statins. Moderate risk is ≥7.5% to <20% in the US, and ≥10% to <20% in England. High risk is ≥20% in both countries.
