# Supplementary material for: Treatment gaps and potential cardiovascular risk reduction from expanded statin use in the US and England
Source: PLoS One. 2018 Mar 21;13(3):e0190688. doi: 10.1371/journal.pone.0190688 (PMC5862405; doi:10.1371/journal.pone.0190688)
Supplement: S3 Table — (DOCX) [file pone.0190688.s003.docx]

**S3 Table. Cardiovascular disease (CVD) events prevented over 10 years (in thousands and rounded to nearest 1000 with 95% confidence intervals) in statin-naïve adults aged 40-75 years without existing CVD and triglyceride levels <400 mg/dL under full treatment in the US using the fasting sample (n=3,010) in NHANES (2007-2012) and accounting for pretreatment LDL-cholesterol levels.**

| Risk group (10-year CVD risk) | Total | 40-59 years | 60-75 years | Men | Women |
| --- | --- | --- | --- | --- | --- |
| Moderate (≥7.5% to <20%) | 790 (645-929) | 338 (274-398) | 451 (362-535) | 515 (417-608) | 278 (223-330) |
| High (≥20%) | 479 (386-576) | 125 (94-162) | 355 (286-425) | 350 (280-423) | 130 (104-157) |
